# N-(3-Oxododecanoyl)-Homoserine Lactone Induces Intestinal Barrier Damage in Piglets via the Lipid Raft-Mediated Apoptosis Pathway

**DOI:** 10.3390/vetsci12030233

**Published:** 2025-03-03

**Authors:** Yang Yang, Xin Zhang, Jin Yang, Ziyan Wu, Junpeng Li, Ruilong Song, Chuang Meng, Guoqiang Zhu

**Affiliations:** 1 College of Veterinary Medicine, Yangzhou University, Yangzhou 225009, China; zhangx41@sbtjt.com (X.Z.); 18255676558@163.com (Z.W.); wei77917weijie@163.com (J.L.); 006018@yzu.edu.cn (R.S.); 2 Jiangsu Co-Innovation Center for Important Animal Infectious Diseases and Zoonoses, and Joint Laboratory of International Cooperation on Prevention and Control Technology of Important Animal Diseases and Zoonoses of Jiangsu, Yangzhou 225009, China; 3 Northern Jiangsu People’s Hospital Affiliated to Yangzhou University, Yangzhou 225009, China; 18051062957@yzu.edu.cn; 4 Jiangsu Key Lab of Zoonosis/Jiangsu Co-Innovation Center for Prevention and Control of Important Animal Infectious Diseases and Zoonoses, Yangzhou University, Yangzhou 225009, China; 006503@yzu.edu.cn

**Keywords:** quorum sensing, OdDHL, IPEC-J2, apoptosis, lipid raft

## Abstract

Quorum sensing (QS) is a process by which bacteria sense their population density and regulate behavior accordingly. QS not only regulates bacterial virulence but also directly influences host cells. Previous studies have shown that QS is strongly associated with piglet intestinal health, but the mechanism is not yet clear. For the first time, we have confirmed in a piglet animal model that OdDHL directly damages intestinal cells in weaned piglets, disrupting the intestinal barrier. We also provide a preliminary exploration of the underlying mechanism of these effects. TUNEL assays confirmed that damage to the piglet intestinal barrier coincided temporally and spatially with dysregulated apoptosis. Lipid rafts, key components of the cell membrane, are involved in many biological processes, including the activation of apoptosis-related proteins. Following the disruption of the lipid raft structure in IPEC-J2 cells, the apoptosis rate under OdDHL stimulation decreased by 50%. These data demonstrate that lipid rafts mediate the attachment of OdDHL to porcine intestinal cells; then, OdDHL induces apoptosis in porcine intestinal cells through the mitochondrial and death receptor pathways, thereby compromising the integrity of the porcine intestinal barrier. This study provides foundational insights into the role of QS in piglet intestinal diseases.

## Simple Summary

Quorum sensing (QS) not only regulates bacterial virulence through signaling molecules, but is also capable of causing direct damage to host cells. Previous studies have found a high correlation between N-acyl homoserine lactone (AHL) of the QS in the piglet intestine and piglet health. In a mouse model, AHL signaling N-(3-oxododecanoyl)-homoserine lactone (OdDHL) induced apoptosis of intestinal cells and damage to intestinal barrier. In a porcine intestinal epithelial cell model, OdDHL also was confirmed to induce apoptosis. The direct role and mechanism of OdDHL in the digestive tract of porcine animal models have not been elucidated. For the first time in a piglet model, we have verified that AHL induces apoptosis and disrupts the intestinal barrier, and we have also verified the protective effect of QS inhibitors. The present study also investigates the central role of cellular lipid rafts in the critical phase in which OdDHL adheres to cells and exerts functions. In cells where lipid rafts were eliminated, the ability of OdDHL adherence to cell surface was significantly reduced, and apoptosis induction was similarly impaired. This result indicates an important role for lipid rafts in relevant processes. This study provides basic data for the antimicrobial application of QSI in pig farming.

## 1. Introduction

The intestine is the site where digestion, nutrient absorption, and microorganism colonization occur in animals [1]. The intestinal microbiota is an indicator of animal health. The intestinal mucosal barrier is an important defense against bacterial invasion and prevents the entry of toxins into the body [2]. The permeability of the mucosal layer increases after intestinal barrier damage. This disrupts the ion balance in the body and opens the gateway for bacteria and toxins to enter the bloodstream, which in turn leads to diseases such as bacteremia and septicemia [3]. The intestinal villi form an essential part of the intestinal mucosal barrier [4]. They promote nutrient absorption, aid in intestinal motility, and resist external threats [5,6].

In pig farming, the incidence of piglet diarrhea is 20–50%. Diarrhea leads to weakened immunity in piglets, high secondary infection rates, and increased mortality, resulting in significant economic losses for the swine industry [7]. Some pathogens use metabolites produced during quorum sensing (QS) to evade immune detection of host intestinal epithelial cells. These pathogens exert their pathogenicity by regulating the expression of virulence factors, ultimately causing diarrhea in the host [8,9,10,11,12,13,14,15,16,17,18]. QS is a unique mechanism of bacterial cell–cell communication via secreted, hormone-like signaling molecules. Most bacteria produce special signaling molecules known as autoinducers (AIs) during growth. Bacteria detect AI signals in the environment. When the AI concentration reaches the threshold level, the expression of virulence genes is triggered, leading to physiological changes. Previous studies have suggested that bacterial QS molecules, especially N-(3-oxododecanoyl)-homoserine lactone (OdDHL) of the N-acyl homoserine lactone (AHL)-mediated QS system, are significant risk factors for intestinal health in low-birth-weight piglets [19].

OdDHL was found to be structurally and functionally similar to mammalian lipid hormones, whereby OdDHL has lipid-soluble and membrane-permeable properties that allow it to bind to mammals and exert its activity, allowing it to enter osteoblasts, epithelial cells, and fibroblasts in a short period of time [15]. OdDHL has a strong pro-apoptotic effect on a wide range of cells, but not all cell types are sensitive [18]. OdDHL disrupts the tight junction proteins and periplasm of intestinal cells, damaging the intestinal barrier [19]. It induces cells to release a variety of pro-inflammatory cytokines and immunomodulatory factors, such as IL-6 and IL-8 from human corneal epithelial cells, and IL-1 and IL-8 from endothelial and mesenchymal cells [20]. However, the function and mechanism of OdDHL in the porcine model have never been resolved.

Various AHL quorum-sensing molecules have been detected in pig feces, including C4-HSL, C6-HSL, C8-HSL, 3OC8-HSL, C10-HSL, 3OC10-HSL, C12-HSL, 3OC12-HSL, and 3OC14-HSL [19]. Low-birth-weight piglets often suffer from intestinal dysfunction and high mortality, resulting in significant economic losses for the swine industry. At 50 days of age, the concentrations of OdDHL and 3OC14-HSL in the feces of low-birth-weight piglets are 2.5- and 2.24-fold higher than those of piglets born with normal weight [19]. In a mouse CT-26 cell model, OdDHL induces apoptosis and disrupts the intestinal epithelial barrier. Apoptosis is a genetically regulated, programmed cell death process [20]. Distinct from necrosis, apoptosis is crucial for maintaining cellular homeostasis [21]. As a properly regulated process, apoptosis serves to clear the “redundant” cells in the body, for example by inducing rapid cell death in intestinal cells after bacterial adhesion to prevent the spread of infection to adjacent cells, which helps the body to eliminate pathogens [22]. However, when apoptosis is disordered, excessive apoptotic activity damages the intestinal epithelial barrier and leads to increased mucosal permeability [23]. As a result, endotoxins and bacteria can enter the systemic circulation and cause systemic infection. OdDHL disrupts the intestinal microbiota, damages the intestinal barrier, and induces systemic inflammation in mice. Fecal microbiota transplantation (FMT) experiments showed that the phenotype of specific pathogen-free (SPF) mice could almost be reproduced in germ-free mice, manifesting as weight loss, colon damage, and changes in serum biochemical markers [24]. Our research group previously found that OdDHL directly stimulates porcine intestinal cells (IPEC-J2), causing changes in cell morphology and promoting cytoskeleton rearrangement [25].

QS molecules regulate the expression of virulence factors in many mammalian intestinal pathogens [8,10,11,25]. In animal husbandry, farmers seek all possible means to mitigate the harm caused by drug-resistant pathogens [26,27,28,29,30]. Antibiotics can kill bacteria, but the selective pressure that antibiotics put on bacteria has led to the emergence of resistance. Nowadays, the global research and development of new antibiotics is very rare, mainly due to the huge cost and long time period. QS can regulate bacterial virulence and biofilm formation, which are directly related to bacterial pathogenicity. QS inhibitors (QSIs) anchor QS regulatory pathway, inhibit bacterial virulence, and reduce bacterial pathogenicity without affecting bacterial survival, which will not cause survival pressure and lead to drug resistance [28]. Antimicrobial agents targeting bacterial QS are gaining attention as alternatives to antibiotics. In aquaculture, antibiotics are limited by the aquaculture environment, and QSI was reported to greatly reduce the infection of *Aeromonas hydrophila* in zebrafish and carp aquaculture [30]. Organic acids and plant extracts functioning as QSIs regulate the expression of efflux pump genes in bacteria and inhibit bacterial invasiveness and infectivity, thus providing a new strategy for combating microbial drug resistance [31,32,33,34,35,36,37]. Extensive evidence has alluded to the involvement of bacterial QS molecules, such as OdDHL, in the damage caused to the host intestinal barrier [38,39]. However, QSIs protect the piglet intestinal barrier from OdDHL damage, although their exact mechanism remains unclear. In our previous studies, OdDHL was found to induce changes in apoptosis-related pathways and the extracellular matrix [25].

This study is the first to explore the mechanism by which OdDHL directly damages the piglet intestinal barrier, confirming that bacterial QS is a significant risk factor for piglet intestinal health in the farming industry. This study also confirms, for the first time in a piglet animal model, the role of QSI in protecting the piglet intestinal barrier. Our findings preliminarily verify the crucial role of lipid rafts in the action of OdDHL on porcine intestinal epithelial cells. We investigated the relationships among QS, pathogens, and hosts, and provide a theoretical basis for using bacterial QS characteristics to aid agricultural farming.

## 2. Results

### 2.1. OdDHL Damages the Intestinal Barrier in Model Piglets

**Figure 1:**
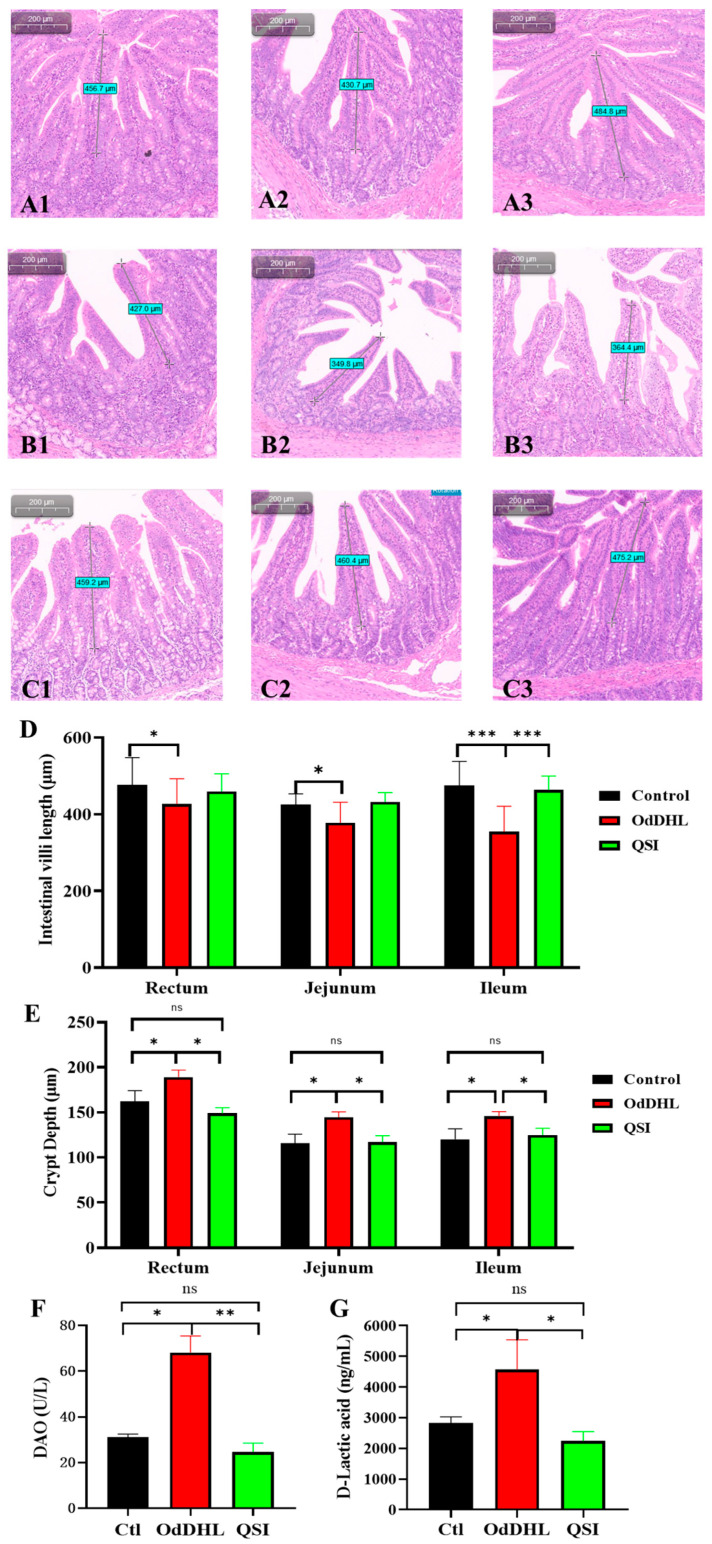
Effect of OdDHL on piglet intestinal barrier. (**A1**–**A3**): Hematoxylin–eosin staining results of duodenum, jejunum, and ileum samples from piglets in the Control group (piglets had free access to normal food and water). (**B1**–**B3**): H&E Staining results of duodenum, jejunum, and ileum samples from piglets in the OdDHL group (piglets were fed 0.5 L of 200 µmol/L OdDHL solution daily, while also having free access to water and regular pig feed). (**C1**–**C3**): H&E Staining results of duodenum, jejunum, and ileum samples from piglets in the QSI group (piglets were fed 0.5 L of 200 µmol/L OdDHL solution daily, while also having free access to water and pig feed with 0.5% (*W*/*W*) AiiA-based QSI added). (**D**) The length of small intestine villi in Control, OdDHL, and QSI groups of piglet model. (**E**) The crypt depth in Control, OdDHL, and QSI groups of piglet model. (**F**) DAO concentration in pig blood in Control (Ctl), OdDHL, and QSI groups of piglet model. (**G**) D-lactic acid concentration in pig blood in Control, OdDHL, and QSI groups of piglet model. *: *p* < 0.05; **: *p* < 0.01; ***: *p* < 0.001; ns: no significance.

H&E-stained intestine tissues were collected from piglets. In the control group, the villi of the duodenum, jejunum, and ileum were dense and elongated, with a long villus length. In the OdDHL group, the villi in the duodenum, jejunum, and ileum appeared shorter and more sparse. No significant difference was observed between the QSI and control groups (Figure 1A–C). The lengths of the small intestinal villi in the duodenum, jejunum, and ileum of the OdDHL group were reduced by 10.5%, 11.2%, and 25.3%, respectively, compared to those in the control group (Figure 1D). The crypt depth in the duodenum, jejunum, and ileum of the OdDHL group were increased by 16.7%, 24.7%, and 21.3%, respectively, compared to those in the control group (Figure 1E). There was no significant difference in the length of the small intestinal villi or crypt depth between the QSI and control groups, demonstrating the protective effect of QSI on the intestinal barrier.

When the intestinal mucosal barrier is mechanically damaged, the levels of DAO and D-lactate increase. In the blood of piglets in the OdDHL group, the DAO and D-lactate concentrations were 2.19- and 1.52-fold higher, respectively, than those in the control group, while there were no significant differences between the DAO and D-lactate levels in the QSI and control groups (Figure 1F,G).

### 2.2. OdDHL Induces Apoptosis in Piglet Intestinal Epithelial Cells In Vivo and In Vitro

**Figure 2:**
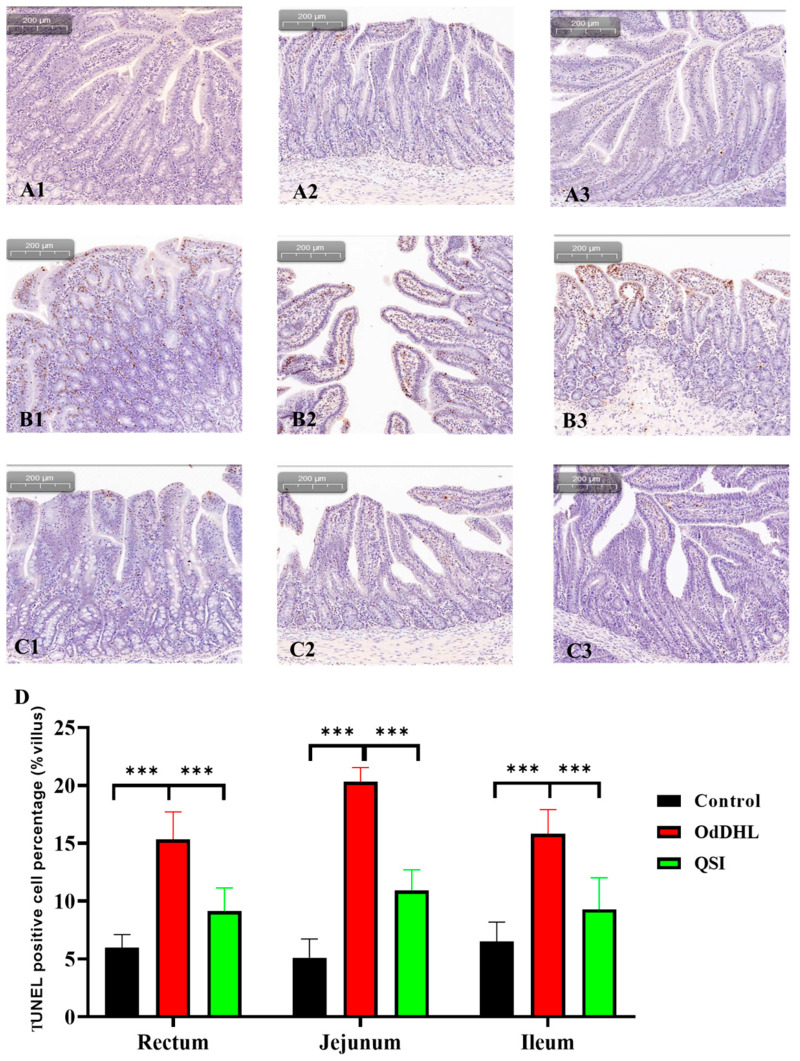
TUNEL test of the intestines of piglets. (**A1**–**A3**): TUNEL results of duodenum (**A1**), jejunum (**A2**), and ileum (**A3**) sample from piglets in Control group (piglets had free access to normal food and water). (**B1**–**B3**): TUNEL results of duodenum (**B1**), jejunum (**B2**), and ileum (**B3**) sample from piglets in OdDHL group (piglet was fed 1 L of 200 µmol/L OdDHL solution daily, while also having free access to water and regular pig feed). (**C1**–**C3**): TUNEL results of duodenum (**C1**), jejunum (**C2**), and ileum (**C3**) sample from piglets in QSI group (piglet was fed 1 L of 200 µmol/L OdDHL solution daily, while also having free access to water and pig feed added with 0.5% (W/W) AiiA-based QSI). (**D**) TUNEL-positive cell percentage, obtained by calculating the proportion of TUNEL-positive cells out of all cells on 10 random villi. ***: *p* < 0.001.

In the animal model, TUNEL was used to detect apoptosis in piglet intestinal tissues. As shown in Figure 2A, only lightly stained brown and blue areas were present in the duodenum, jejunum, and ileum of the control group, indicating normal apoptosis occurring as part of the physiological process. In contrast, in the OdDHL group, extensive and overlapping brown and blue areas were observed in the cell nuclei, indicating a significant increase in the apoptosis rate of intestinal cells (Figure 2B,D). In the QSI group, only normal apoptotic cells were observed, with no significant upregulation in apoptosis, thus confirming the protective effect of QSI on the small intestine of piglets (Figure 2C).

**Figure 3:**
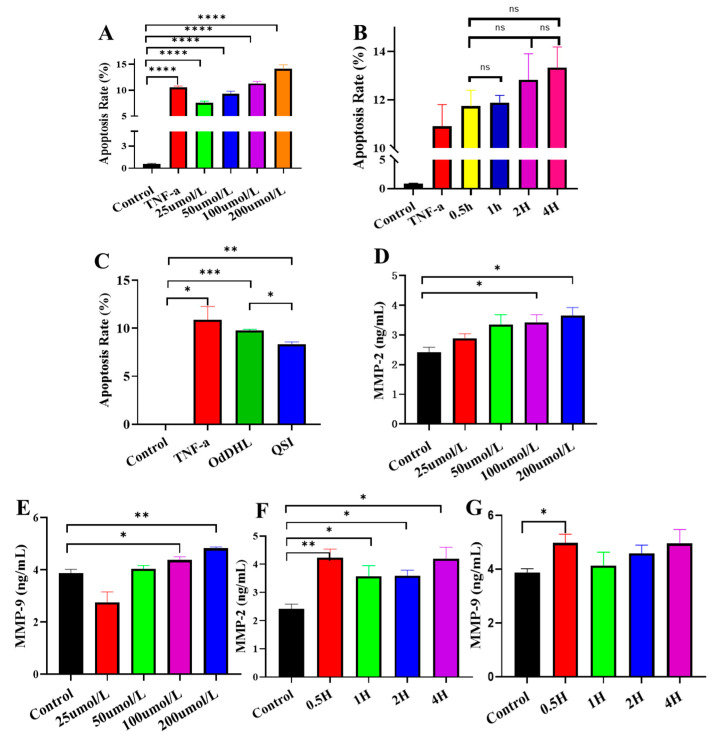
Cell apoptosis-related parameter detection. (**A**) Apoptosis rate under different concentrations of OdDHL on IPEC-J2 cell; apoptosis rate is the proportion of the number of apoptotic cells out of a given total number of cells in flow cytometry. (**B**) Apoptosis rate under different time of OdDHL on IPEC-J2 cell. (**C**) QSI quenching of the apoptosis rate from OdDHL; in Control group, PBS was added into cells; TNF-α was added as apoptosis inducer. (**D**) Effect of different concentrations of OdDHL on MMP-2 expression in IPEC-J2. (**E**) Effect of different concentrations of OdDHL on MMP-9 in IPEC-J2. (**F**) Effect of different duration of OdDHL on MMP-2 in IPEC-J2. (**G**) Effect of different duration of OdDHL on MMP-9 in IPEC-J2. *: *p* < 0.05; **: *p* < 0.01; ***: *p* < 0.001; ****: *p* < 0.0001; ns: no significance.

Subsequently, in the porcine small intestinal epithelial cell model (IPEC-J2), different concentrations of OdDHL (25 µmol/L, 50 µmol/L, 100 µmol/L, 200 µmol/L) were applied, resulting in apoptosis rates of 7.41%, 9.52%, 11.5%, and 13.71%, respectively (Figure 3A). Compared to the negative control, the apoptosis rate increased dose-dependently, with a significant uptick in apoptosis following OdDHL induction. However, the correlation between the apoptosis rate and OdDHL (200 µmol/L) exposure time was weak and statistically insignificant (Figure 3B). Therefore, in subsequent experiments, the OdDHL concentration was set at 200 µmol/L, and the exposure time was 2 h.

QSIs hydrolyze quorum-sensing signaling molecules to effectively lower the apoptosis rate of piglet intestinal epithelial cells. The results show that in the QSI group, the IPEC-J2 apoptosis rate decreased by 15.3%, indicating a significant reduction in OdDHL-induced apoptosis (Figure 3C).

MMP-2 and MMP-9 are closely associated with apoptosis [40]. OdDHL effectively and dose-dependently upregulated the expression of MMP-2 and MMP-9 (Figure 3D,E). The expression of MMP-2 and MMP-9 was weakly correlated with the duration of OdDHL exposure (Figure 3F,G).

### 2.3. OdDHL Significantly Regulates the Expression of Apoptosis-Related Proteins

**Figure 4:**
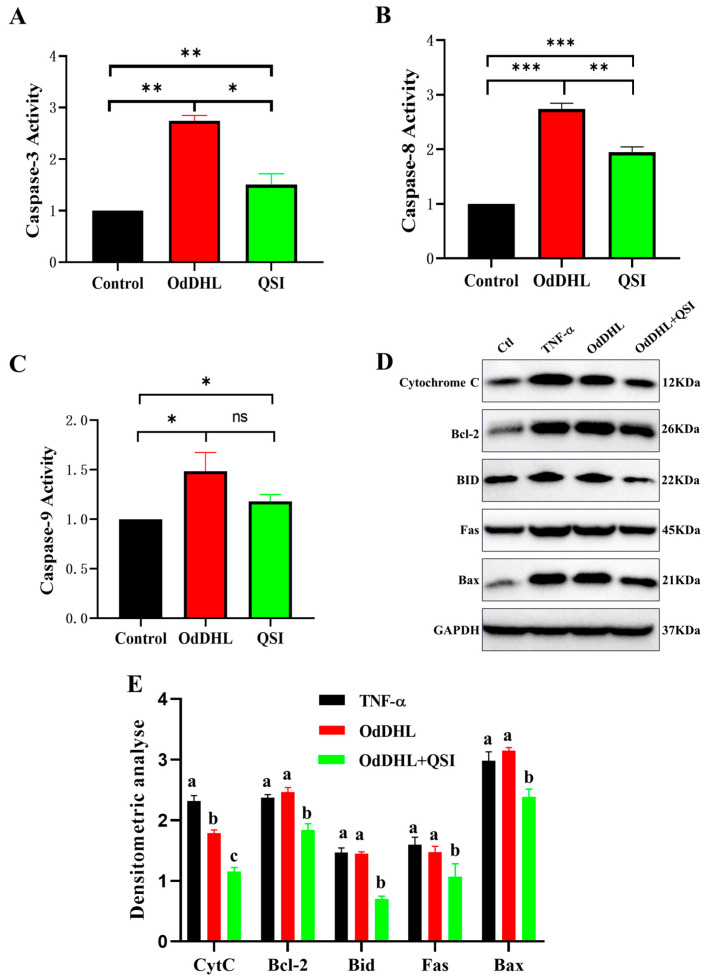
Caspase activity test and apoptosis-related protein expression level detection. (**A**) Caspase-3 activity in Control, OdDHL, and QSI groups of cell model. In Control group, PBS was added into cells. In OdDHL group, 200 µmol/L OdDHL were added into each cell well. In the QSI team, a mixture of OdDHL and QSI was added into cell wells. (**B**) Caspase-8 activity in Control, OdDHL, and QSI groups of cell model. (**C**) Caspase-9 activity in Control, OdDHL, and QSI groups of cell model; caspase activity was determined by calculating the multiplicity of OD_450_ values of samples/OD450 values of negative control. (**D**) Expression of apoptosis related proteins by Western blot. TNF-α was added as apoptosis inducer. (**E**) Densitometric analyses of apoptosis related proteins. Each value represents the mean ± SD (*n* = 3) considering the control sample value as 1. Different letters indicate significant differences between treatments. *: *p* < 0.05; **: *p* < 0.01; ***: *p* < 0.001; ns: no significance. The original images of the Western blot are published as supplementary material.

The caspase family of proteins plays an essential role in apoptosis. Caspase-3 and -8 are involved in the death receptor-mediated apoptosis pathway, whereas caspase-3 and -9 are involved in the mitochondrial pathway of apoptosis [41]. The results showed that OdDHL significantly increased the activities of caspases 3, 8, and 9, whereas the stimulatory effect of OdDHL on these caspases was significantly inhibited after QSI treatment (Figure 4A–C).

Western blotting (Figure 4D,E) revealed that the expression of the apoptosis-related proteins CytC, Bid, BAX, Bcl-2, and FASCD95 was relatively low under normal conditions. After stimulation with the apoptosis inducer TNF-α, the expression of CytC, BAX, Bcl-2, and FASCD95 was significantly upregulated. When OdDHL was co-incubated with cells, the expression of BAX, Bcl-2, and FASCD95 was significantly increased, whereas no significant difference was observed in the expression of CytC and Bid. The results of the QSI group demonstrated that QSI effectively inhibited the stimulatory effect of OdDHL on the expression of apoptosis-related proteins.

### 2.4. Lipid Rafts Are Closely Related to OdDHL-Induced Apoptosis in Porcine Intestinal Cells

**Figure 5:**
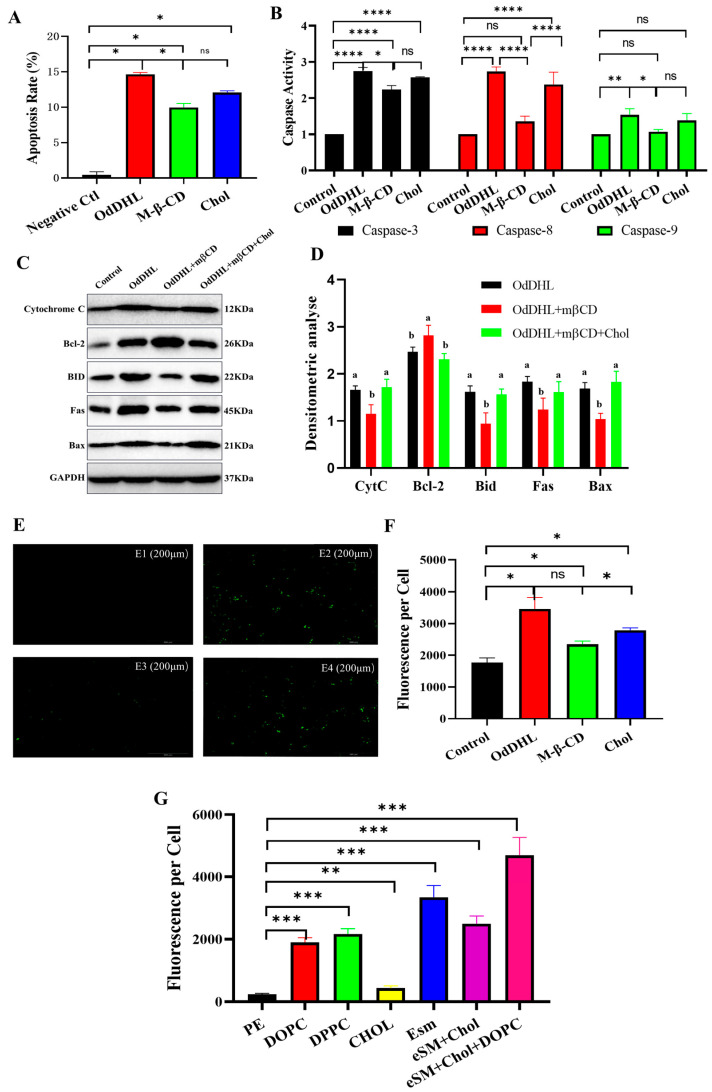
Detection of OdDHL-related parameters after removal of lipid rafts from IPEC-J2. (**A**) Detection of cell apoptosis rate. In negative Control group, PBS was added into cells. In positive control group, TNF-α was added as apoptosis inducer. In OdDHL group, 200 µmol/L OdDHL was added into each cell well. In M-β-CD group, to remove lipid rafts from IPEC-J2 surface, 2 mg/mL M-β-CD was added for 1 h incubation. In Chol group, cells were incubated with 400 µg/mL cholesterol for 1 h before M-β-CD treatment. (**B**) Detection of caspase activity. (**C**) Expression level of apoptosis-related proteins. (**D**) Densitometric analyses of apoptosis-related proteins. Each value represents the mean ± SD (n = 3) considering the control sample value as 1. Different letters indicate significant differences between treatments. (**E**) Fluorescence microscope observation after co-incubation with FITC-OdDHL conjugates: E1—negative control; E2—OdDHL group; E3—M-β-CD group; E4—Chol group. (**F**) Fluorescence intensity of individual cells in flow cytometry. (**G**) Affinity of OdDHL to different lipids. *: *p* < 0.05; **: *p* < 0.01; ***: *p* < 0.001; ****: *p* < 0.0001; ns: no significant.

After M-β-CD treatment, the mean fluorescence intensity from CTB (Cholera Toxin Subunit B)-FITC (Absin, Shanghai, China) binding to GM1 decreased by 40%, indicating successful disruption of lipid raft on IPEC-J2. In the Chol group, fluorescence intensity was increased by 23% compared with M-β-CD group, which indicated that the lipid rafts had recovered to some extent. TNF-α, used as a positive control, induced an average apoptosis rate of 10.23%, but a rate of 14% in the OdDHL group. However, after lipid rafts in intestinal epithelial cells were removed with M-β-CD, the rate of OdDHL-induced apoptosis decreased by approximately 50%. In the Chol group, the addition of cholesterol partially restored the lipid rafts, and the apoptosis rate increased by approximately 12% compared to that in the M-β-CD group (Figure 5A).

In the M-β-CD group, the ability of OdDHL to induce caspase expression was similarly tested. After lipid raft removal, the activities of caspases 3, 8, and 9 all showed varying degrees of decline. In the Chol group, the apoptosis rate was increased (Figure 5B).

Western blotting experiments suggested that after lipid raft removal, IPEC-J2 cells showed lower CytC, Bid, BAX, and FASCD95 expression compared to those in the OdDHL-treated group, while Bcl-2 expression increased. These results indicate that lipid rafts participate in OdDHL-induced apoptosis in IPEC-J2 cells. In the Chol group, the expression of apoptosis-related proteins increased (Figure 5C,D).

FITC-OdDHL conjugates were co-incubated with IPEC-J2 cells. Fluorescence microscopy revealed that OdDHL effectively adhered to IPEC-J2 cells, with intense green fluorescence observed. However, after removing the lipid raft structure from the cells, the intensity of green fluorescence decreased significantly, proving that lipid rafts are directly involved in OdDHL adherence to cells. In the Chol group, a significant increase in green fluorescence was observed after partial lipid raft replenishment (Figure 5E).

Flow cytometry was used to quantify the fluorescence intensity of individual cells in each group. In the M-β-CD group, the fluorescence intensity of single cells displaying green fluorescence (i.e., cells that adsorbed OdDHL) was significantly lower than that in the OdDHL group by approximately 32.71%. The Chol group showed significantly higher fluorescence intensity than the M-β-CD group (approximately 18.72% higher, Figure 5F).

FITC-OdDHL conjugates were used to measure the affinity and adsorption of OdDHL toward various lipids that comprise lipid rafts to determine the lipids that play a major role in OdDHL adherence and its action on porcine intestinal epithelial cells. Artificially synthesized lipids such as PE (phosphatidylethanolamine) were used as negative controls. The results show that OdDHL has a low affinity for simple lipids, particularly for PE and Chol (cholesterol). However, compared to the negative controls, the affinity of OdDHL toward the SM (sphingomyelin) and Chol/DOPC (1,2-dioleoyl-sn-glycero-3-phosphocholine) mixture [42] (commonly used to simulate lipid raft structures) was significantly higher (19.9 times higher, Figure 5G).

## 3. Discussion

Using weaned piglets fed purified QS signaling molecules (OdDHL) and QSI (AiiA) as an animal model, we explored the mechanism by which OdDHL damages the intestinal barrier and the protective effect of QSI on it. Under the stimulation of OdDHL, the small intestinal villi of piglets shrink, become sparser, and shorten in length. When the intestinal mucosal barrier is mechanically damaged, the levels of two key indicators, DAO and D-lactate, increase [43]. Our results revealed that, after administering OdDHL to piglets via drinking water, the peripheral blood levels of DAO and D-lactate significantly increased, confirming that the QS signaling molecule OdDHL can directly damage the intestinal mucosal barrier. Adding QSI to the feed effectively protected the intestinal barrier from OdDHL-induced damage.

A TUNEL assay was designed to detect apoptotic cells [44], and it revealed that following OdDHL treatment, large areas of apoptotic cells were clearly observed in the duodenum, jejunum, and ileum of piglets, whereas QSI effectively inhibited apoptosis. This finding demonstrates, for the first time, that QS molecules induce unregulated intestinal cell apoptosis in a piglet model and that OdDHL-induced apoptosis is the direct cause of intestinal barrier damage triggered by QS. Current research confirms that apoptosis can be categorized into exogenous and endogenous pathways. The exogenous pathway is regulated by stimulation of tumor necrosis factor receptor (TNFR) superfamily members. The endogenous pathway is characterized by mitochondrial outer membrane permeabilization and mitochondrial cytochrome c release, leading to the assembly of a caspase activation complex between caspase-9 and apoptotic bodies. Pathogen infection causes structural damage and loss of function in host cells and stimulates the host’s recognition and immune response. Pathogen-induced apoptosis enhances or diminishes pathogenicity. Apoptosis is an active physiologic death process mediated by multiple genes. Apoptosis is a highly regulated energy-dependent cell death and is an important pathway for host to resist external infections. Apoptosis occurs as a result of a combination of pro- and anti-apoptotic genes [45,46,47,48,49].

Subsequently, we explored the mechanism underlying OdDHL-induced apoptosis in intestinal cells using an IPEC-J2 cell model. Flow cytometry using annexin-FITC assay showed that OdDHL directly induced apoptosis in IPEC-J2 cells. Under OdDHL induction, the activities of caspases 3, 8, and 9 were significantly increased, as determined by ELISA. However, QSI significantly inhibited the stimulatory effect of OdDHL on the activities of caspases 3, 8, and 9. During the early stages of apoptosis, Caspase-3 is activated to cleave cytoplasmic and nuclear substrates, leading to apoptosis. Caspase-3 and -8 are involved in the death receptor pathway of apoptosis, whereas caspase-3 and -9 are related to the mitochondrial apoptosis pathway [22]. These results suggest that OdDHL induces apoptosis through both the death receptor and mitochondrial pathways. The Western blot results showed that following OdDHL treatment, the expression of the apoptosis-related proteins CytC, BAX, and FASCD95 increased significantly, whereas QSI significantly inhibited the changes in the expression levels of apoptosis-related proteins. These results demonstrate that OdDHL, a bacterial QS molecule, induces apoptosis through multiple pathways and that its ability to induce apoptosis is lost after its degradation by QSI. MMPs degrade extracellular matrix and are closely related to apoptosis [40]. We demonstrated that the concentration of MMPs in IPEC-J2 cells changed depending on the dose of OdDHL applied. Therefore, OdDHL may indirectly influence apoptosis by affecting MMPs.

The cell membrane is a signaling platform for intercellular communication. Various proteins on the cell membrane can bind to extracellular signaling molecules and transmit signals to cells, inducing specific biological effects. Lipid rafts are an important component of cell membranes and are involved in many biological processes, including transmembrane signal transduction, apoptosis, Chol transport, and protein sorting during endocytosis and exocytosis [50,51]. Lipid rafts are cell membrane structures composed of Chol, glycosylphosphatidylinositol-anchored proteins, glycosphingolipids, SM, and unsaturated acyl chain phospholipids. Many studies have reported that lipid rafts are involved in cell invasion by non-enveloped viruses, such as rotavirus, rhinovirus, and enterovirus [52]. Additionally, lipid rafts play a role in the colonization of host cells by pathogens such as *Vibrio cholerae* and Enteropathogenic *Escherichia coli* [53]. The spatial structure of acyl homoserine lactones provides them with both hydrophilic and hydrophobic properties, but it is not clear whether they adhere to host intestinal cells via lipid rafts.

Because lipid rafts contain high Chol concentrations, they exhibit a liquid-ordered state that is between the liquid crystalline and gel phases. Removal of Chol with M-β-CD disrupts the phase of lipid rafts [54], thereby impairing their biological functions. In this study, FITC-OdDHL conjugates were applied to IPEC-J2 cells, and fluorescence microscopy and flow cytometry were used to qualitatively and quantitatively observe the green fluorescence intensity of the cells in each group. The results showed that after M-β-CD treatment, the number of OdDHL molecules adsorbed to the surface of IPEC-J2 cells was significantly reduced, whereas Chol supplementation restored the ability of the cells to adsorb FITC-OdDHL molecules. Flow cytometry was used to quantitatively analyze the fluorescence intensity of individual cells in each group. The results showed that after M-β-CD treatment, the ability of individual IPEC-J2 cells to adsorb OdDHL was significantly weakened. These results demonstrate that lipid rafts mediate the adsorption of OdDHL molecules to cells and that disrupting their structure significantly reduces the ability of cells to adsorb OdDHL molecules.

We further explored whether the adsorption of OdDHL to lipid rafts is due to its affinity for specific lipids or requires the mediation of a complete lipid raft structure. Various lipids found in lipid rafts were tested, with PE (a synthetic lipid, not present in lipid rafts) used as the control. FITC-OdDHL conjugates were applied to DPPC, PE, DOPC, Chol, and SM. The results show that OdDHL exhibited a much lower affinity for individual lipids than simulated lipid raft structures (composed of SM, Chol, and DOPC) [42]. This confirms that mediation by specific lipid raft structures is required for OdDHL to exert its damaging effect on the intestinal barrier.

The QS system is closely related to bacterial virulence. In the context of the complete ban on antibiotic use in farming in China since 2020, antibacterial drugs targeting bacterial QS have gained increasing attention as alternatives to antibiotics [31,33,34,55]. Since the discovery of bacterial QS in the 1990s, research on its application in the population-level control of bacterial traits has deepened, and it has been closely linked to the synthesis of virulence factors in various pathogenic bacteria. QSIs are used to interfere with QS signals and suppress the expression of virulence genes. In this way, bacterial pathogenicity can be reduced without creating selective pressure, thereby preventing the rapid emergence of antibiotic resistance. Using a piglet model, we demonstrate for the first time that the bacterial QS molecule OdDHL is mediated by lipid rafts and induces intestinal cell apoptosis through both the death receptor and mitochondrial pathways, causing damage to the piglet intestinal barrier, while QSIs effectively protect the piglet intestinal barrier.

In conclusion, this study provides a preliminary explanation for specific roles of QS and QSI and explores the mechanism. To our knowledge, this is the first qualitative piglet model experiment to validate the effect of QS and QSI on the intestinal barrier. Although the research has a degree of novelty, the authors recognize the limitation of the experimental data. For the purpose of the qualitative experiment, only two piglets were arranged in each group in this manuscript. The authors believe that the data obtained from this number of experimental animals have been able to adequately qualitatively validate the function of QS, and that the results from piglets are in complete accordance with the results of cell model that have been repeated enough times. However, the authors also acknowledged that the insufficient number of experimental animals limited the continuation of deeper digging into the relevant conclusions. We believe that this study provides preliminary data on the function and mechanism of QS and QSI in the piglet model, and also believe that the current data can provide inspiration for subsequent replicated animal experiments on adequate piglet numbers, which could thoroughly elucidate the potential function of QS in pig farming and the prospect of QSI application, and could provide theoretical support for the prevention and control of infectious diseases in livestock and poultry under antibiotic restriction policies.

## 4. Materials and Methods

### 4.1. Ethics Statement

All animal experiments were conducted in compliance with the Guidelines for the Care and Use of Laboratory Animals issued by the Ministry of Science and Technology of the People’s Republic of China. The animal experiments were approved by the Animal Welfare and Ethics Committee of Yangzhou University (Approval Number: 202103003) and were conducted in compliance with the ethical standards of animal welfare and the Jiangsu Provincial Animal Management Committee. The experiments were performed at the Animal Experiment Center of the College of Veterinary Medicine, Yangzhou University.

### 4.2. Experimental Animals

Six weaned piglets (Duroc × [Yorkshire × Landrace]) used in this study were purchased from Jiangsu Gangwan Agricultural Science & Technology Group, Yangzhou, China (average initial body weight: 7.0 ± 1.0 kg, age: 28 ± 1 days). The piglets were divided into the control, OdDHL, and QSI groups, with two piglets per group. The authors performed several repetitions of a pig epithelial cell model to determine a suitable working concentration (200 μM) of OdDHL [25]. In order to avoid the stress associated with gavage administration, the dosing drinkers were used to precisely control the amount of solution that piglets drank freely. The control group of piglets had free access to regular food and water. In the OdDHL and QSI groups, each piglet was fed 0.5 L of 200 µmol/L OdDHL (Changzhou Wanyao Biotechnology Co., Changzhou, China) solution daily through rationed waterers. After drinking 0.5 L of solution, piglets were able to drink regular water freely. The piglets in the OdDHL group had free access to regular pig feed. In the QSI group, 0.5% (W/W) AiiA-based QSI (1000 U/g, Beijing Challenge Agricultural Technology Co., Beijing, China) was added to the feed. The pigsty was cleaned daily and disinfected regularly to remove any external influences. After 5 days of feeding, blood was collected from the anterior vena cava of the piglets and stored at 4 °C. The piglets were euthanized with a sodium pentobarbital overdose of 40 mg/kg body weight, followed by exsanguination. The duodenum, jejunum, and ileum of piglets from each group were collected and fixed in 4% paraformaldehyde (Yuanye Bio-Technology, Shanghai, China).

### 4.3. Cell Lines

The IPEC-J2 (intestinal porcine enterocyte) cell line (DSMZ ACC 701) was obtained from the Faculty of Veterinary Medicine in the Department of Microbiology at Yangzhou University [56]. The IPEC-J2 cells were cultured in RPMI 1640-F12 medium supplemented with 10% neonatal bovine serum at 37 °C with 5% CO_2_.

In apoptosis-related experiments, TNF-α (Beyotime, Shanghai, China) was added to IPEC-J2 cells as a positive control for induced apoptosis. In the OdDHL group, different concentrations of OdDHL (25 µmol/L, 50 µmol/L, 100 µmol/L, 200 µmol/L) were added to each well and co-incubated with IPEC-J2 for 0.5 h [25]. Furthermore, 200 µmol/L OdDHL was added for apoptosis rate detection in different OdDHL exposure times (0.5 h, 1 h, 2 h, 4 h). In the QSI treatment, a mixture of OdDHL (200 µmol/L) and QSI (10 μg/mL) was added to each well for incubation. PBS was used as a control. The experiments were repeated three times.

In the lipid raft experiments, for the M-β-CD group, 2 mg/mL of M-β-CD was added to remove lipid rafts from the IPEC-J2 surface, followed by 1 h of incubation. In the cholesterol group, cells were incubated with 400 µg/mL cholesterol for 1 h before M-β-CD treatment to reduce potential damage to the lipid rafts. The ganglioside GM1 is a commonly used raft marker. In order to validate the disruption and recovery of the lipid raft structure, the efficiency was determined by detecting fluorescent CTB-FITC binding to GM1 using flow cytometry [54]. Decreased or increased fluorescence intensity indicated disruption or recovery of lipid raft on cells.

### 4.4. Piglet Intestinal Hematoxylin and Eosin (H&E) Staining

The intestinal tissues were fixed with paraformaldehyde, dehydrated, embedded in paraffin, and sectioned using a microtome (with a section thickness of 5 μm) [57]. After the tissue sections were placed on slides and air-dried, they were stained using a H&E staining kit (Beyotime, Shanghai, China) and observed under an Olympus CK43 microscope (Olympus, Tokyo, Japan). For each intestinal segment, the 10 longest, straight-traced, and well-extended small intestinal villi were selected from each slice. The length of selected small intestinal villi (from the base of the villus to the tip of the villus) and the depth of the crypt (from the base of the crypt to the base of the villus) were measured. The mean values of villi length and crypt depth were calculated.

### 4.5. Enzyme-Linked Immunosorbent Assay (ELISA)

Serum levels of diamine oxidase (DAO), D-lactate, and cell levels of matrix metalloproteinase (MMP)2, MMP9, caspase 3, caspase 8, and caspase 9 were measured using ELISA kits (Mlbio, Shanghai, China) as specified in the manufacturer’s instructions. It mainly consists of steps such as addition of sample, incubation, washing, addition of substrate, and termination. The absorbance at 450 nm of each sample was measured using a microplate reader (Epoch, BioTek, Winooski, VT, USA).

For blood samples (for DAO and D-lactate detection), the precipitated serum was used for subsequent ELISA. For cell samples (for MMP2, MMP9, caspase 3, caspase 8, and caspase 9 measurement), the adherent cells were digested by trypsin. Cells were washed with PBS, then an appropriate amount of lysate solution for resuspension was added. Cells were lysed in an ice bath for 30 min, then centrifuged at 12,000 rpm for 10 min at 4 °C, and the supernatant was aspirated for subsequent experiments.

For DAO, D-lactate, and MMPs, a standard curve is constructed using the standards in the kit so that the content can be calculated. Caspase Activity was determined by calculating the multiplicity of OD_450_ values of samples/OD_450_ values of negative control, in which group PBS was added into cells. The experiments were repeated three times.

### 4.6. TUNEL Assay for Detecting Piglet Intestinal Apoptosis

The TUNEL assay was performed according to the manufacturer’s instructions (Beyotime, Shanghai, China). Briefly, the paraffin-embedded tissue sections were washed twice with phosphate-buffered saline (PBS), and excess water was removed. Each sample was mixed with 50 µL of the TdT enzyme-reaction solution, covered with a cover slip, and incubated at 37 °C in the dark for 60 min. After three washes with PBS, excess solution was removed, and 50 µL of streptavidin-HRP working solution was added, followed by incubation at 37 °C in the dark for 30 min. After three more washes with PBS, 100 µL of DAB (diaminobenzidine)-HRP working solution was added for color development at room temperature for 10 min. The sections were washed with PBS, air-dried, and observed under a microscope. Ten villi were randomly selected from each section and the proportion of TUNEL-positive cells in each villus was counted.

### 4.7. Annexin-FITC Measurement of Apoptosis Rate

Apoptosis assessment by Annexin-FITC was performed as specified in the manufacturer’s instructions (Beyotime, Shanghai, China). TNF-α-induced apoptosis was used as a positive control. Pre-treated cells were washed with pre-chilled PBS, re-suspended, and counted. A total of 50,000 cells were incubated with 195 µL of Annexin-FITC binding solution, followed by 5 µL of Annexin-FITC stain. Then, 5 µL of propidium iodide (PI) was added and mixed, and the cells were incubated at room temperature in the dark for 20 min. The apoptosis rate of each experimental group was measured using a flow cytometer (Cytoflex, Beckman Colter, CA, USA). The experiments were repeated three times.

### 4.8. Western Blot

Western blotting was conducted to detect the expression levels of CytC, Bid, BAX, Bcl2, and FASCD95, following a method described in a related study [57]. Treated cells were washed three times with PBS. Subsequently, 200 µL of cell lysis buffer was added to the cells. After conducting SDS-PAGE and electro transfer, primary antibodies (Wuhan Sanying Biotechnology Co., Wuhan, Hubei, China) were added (Anti-CytC antibody at 1:1000, Bid at 1:1000, BAX at 1:1000, Bcl2 at 1:500, FASCD95 at 1:500, and GAPDH at 1:2000). After the secondary antibody (goat anti-mouse IgG HRP at 1:2000) was added, detection was carried out with a BeyoECL Plus Kit (Beyotime, Shanghai, China). Protein bands were quantificated by densitometric analysis (ImageJ v.1.49, National Institutes of Health, Bethesda, MD, USA). The experiments were repeated three times.

### 4.9. OdDHL Adsorption to Lipid Rafts

OdDHL-FITC conjugates (Cayman Chemical, Ann Arbor, MI, USA) were added to treated cells in each group and incubated for 1 h to observe the affinity of OdDHL for lipid rafts. PBS was used to wash away unbound OdDHL-FITC, and fluorescence microscopy was used for qualitative observation.

The cells from each group were digested with trypsin (without EDTA) and washed with PBS to remove unbound OdDHL-FITC. The fluorescence intensity of each group of cells was quantified using flow cytometry to assess the affinity of OdDHL. The experiments were repeated three times.

### 4.10. OdDHL Lipid Affinity Detection

DOPC (1,2-dioleoyl-sn-glycero-3-phosphocholine), DPPC (1,2-dipalmitoyl-sn-glycero-3-phosphorylcholine), SM (sphingomyelin), Chol (cholesterol), and PE (phosphatidylethanolamine) were prepared at a concentration of 0.01 g/L. Lipids were purchased from Shanghai Aladdin Biochemical Technology Co. (Shanghai, China).

Briefly, 50 µL of each lipid solution was added to a 96-well plate, with four wells containing each lipid solution. For the DOPC/DPPC group, the two lipids were mixed at a 1:1 ratio. For the SM/Chol/DOPC group, the three lipids were mixed at a 6:3:1 ratio to simulate lipid raft formation [42]. After loading the samples, the plate was placed in an incubator at 37 °C for sufficient drying time. Subsequently, 50 µL of OdDHL-FITC was added to each well, incubated at room temperature for 30 min, and washed with PBS. Finally, 20 µL of PBS was added to each well, and the fluorescence intensity was measured using a BioTek Synergy HTX microplate reader (BioTek, Winooski, VT, USA) at an emission wavelength of 485 nm and excitation wavelength of 520 nm. The experiments were repeated three times.

### 4.11. Statistical Analysis

All experimental data were analyzed by unpaired Student’s *t*-test using Prism 5.0 software (GraphPad Inc., San Diego, CA, USA). *p* < 0.05 was considered to be significant statistically. Error bars in the figures represent the standard deviation of the dataset (mean ± standard deviation). * *p* < 0.05, ** *p* < 0.01, *** *p* < 0.001, **** *p* < 0.0001.

## 5. Conclusions

This study verified that bacterial OdDHL signal may disrupt the host intestinal barrier by OdDHL-induced apoptosis. The mechanism of OdDHL-induced apoptosis in a piglet model was preliminarily explored. Furthermore, QSI effectively antagonized this OdDHL-induced intestinal barrier damage. Lipid rafts are intimately involved in the process of OdDHL adsorption to the cell surface and thus play an important role in OdDHL-mediated apoptosis.

## Data Availability

All datasets generated for this study are included in the article.

## Supplementary Materials

The following are available online at https://www.mdpi.com/article/10.3390/vetsci12030233/s1, Figure S1: the original image of the Western blot from Figure 4D, Figure S2: the original image of the Western blot from Figure 5C, Figure S3: flow cytometry diagram in Figure 5F, Figure S4: flow cytometry diagram in Figure 3B, Figure S5: flow cytometry diagram in Figure 3C, Figure S6: flow cytometry diagram in Figure 5F.
