# Supplementary material for: N-(3-Oxododecanoyl)-Homoserine Lactone Induces Intestinal Barrier Damage in Piglets via the Lipid Raft-Mediated Apoptosis Pathway
Source: Vet Sci. 2025 Mar 3;12(3):233. doi: 10.3390/vetsci12030233 (PMC11946647; doi:10.3390/vetsci12030233)
Supplement: Supplementary file 1 [file vetsci-12-00233-s001.zip › vetsci-3449109-supplementary.pdf]

Figure S1

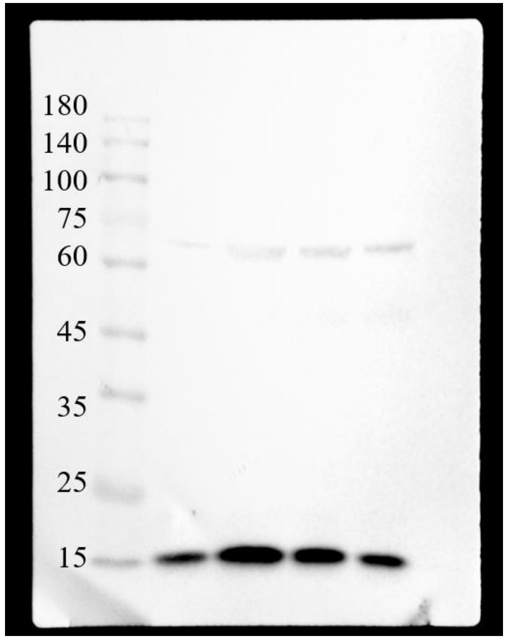

Cyt C (12KD) in Figure 4D

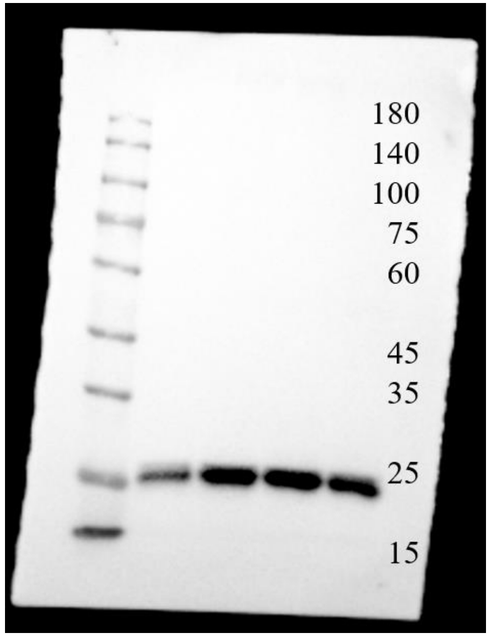

Bcl-2 (26KD) in Figure 4D

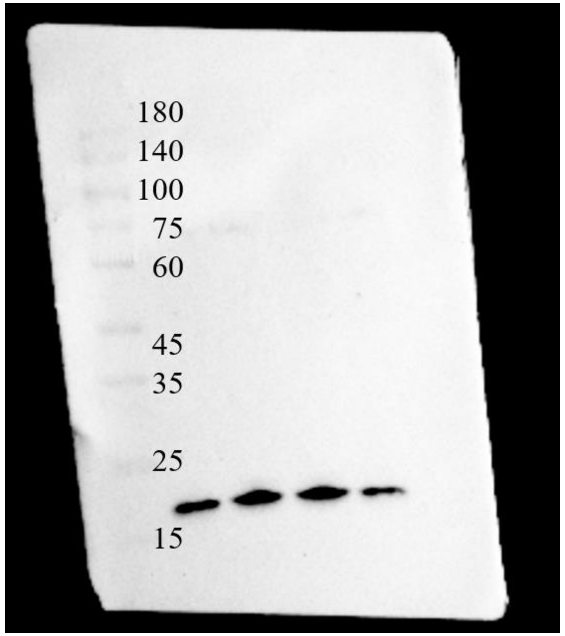

Bid (22KD) in Figure 4D

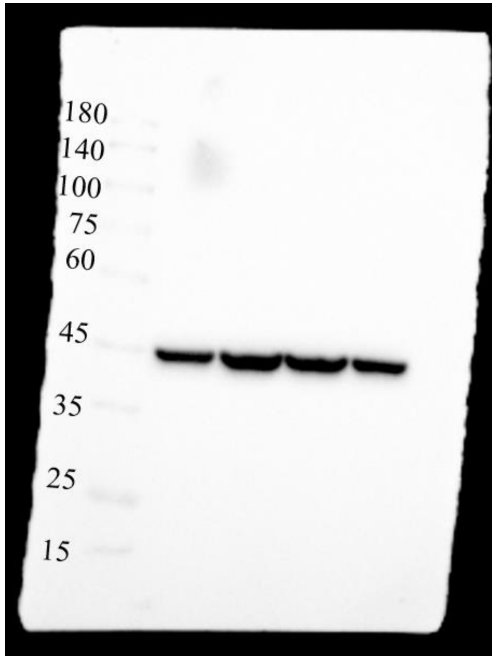

Fas (45KD) in Figure 4D

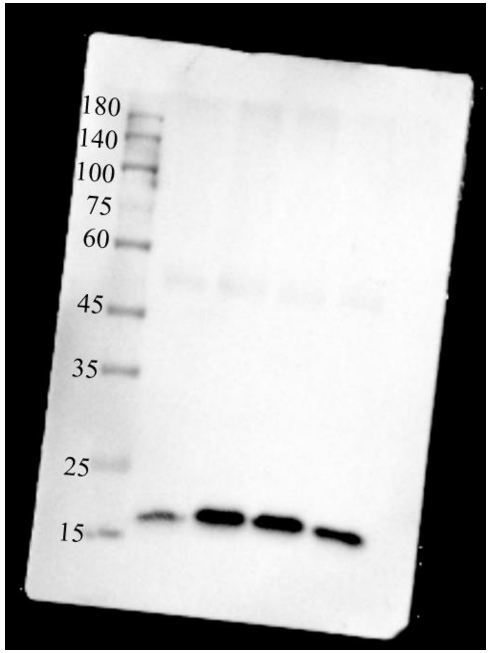

Bax (21KD) in Figure 4D

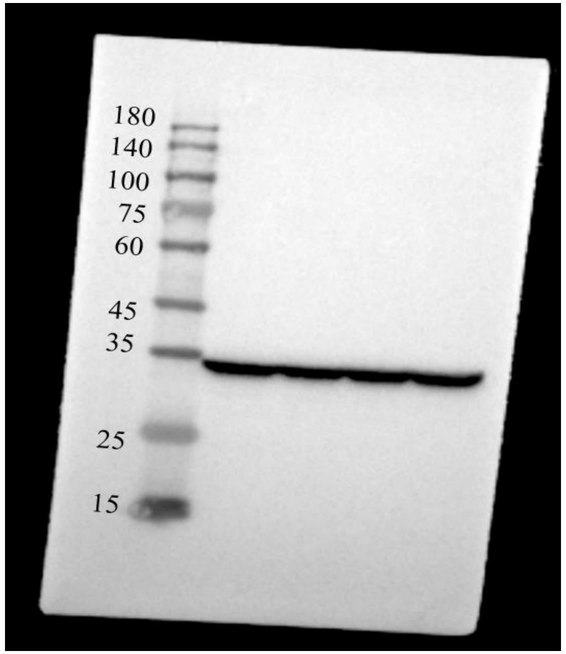

GAPDH (37KD) in Figure 4D

Figure S2

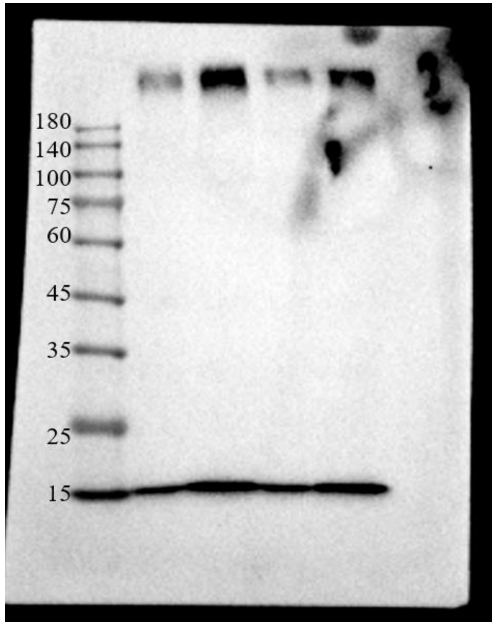

Cyt C (12KD) in Figure 5C

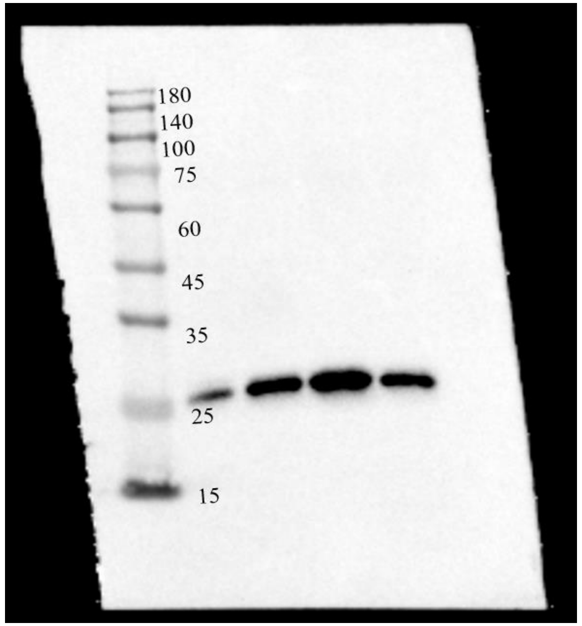

Bcl-2 (26KD) in Figure 5C

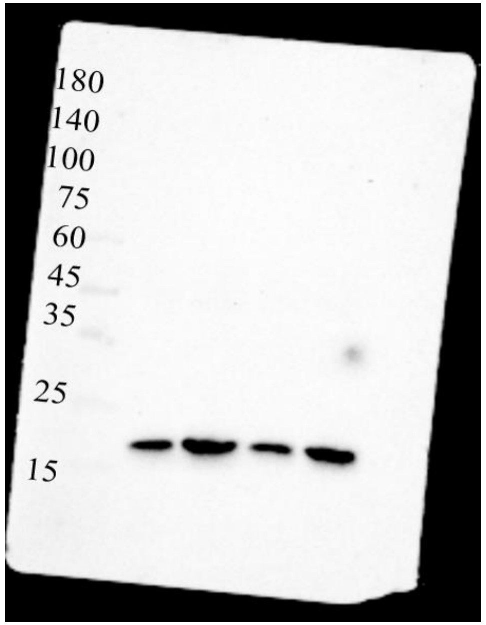

Bid (22KD) in Figure 5C

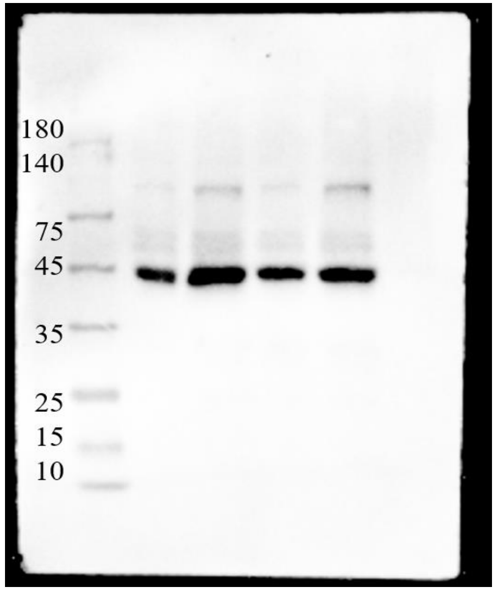

Fas (45KD) in Figure 5C

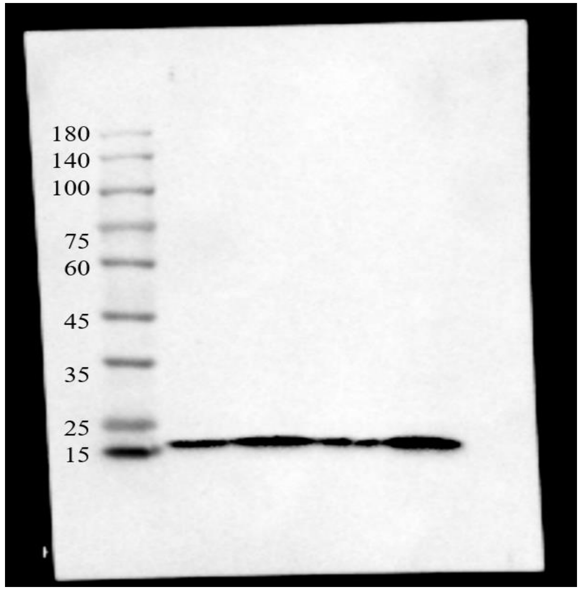

Bax (21KD) in Figure 5C

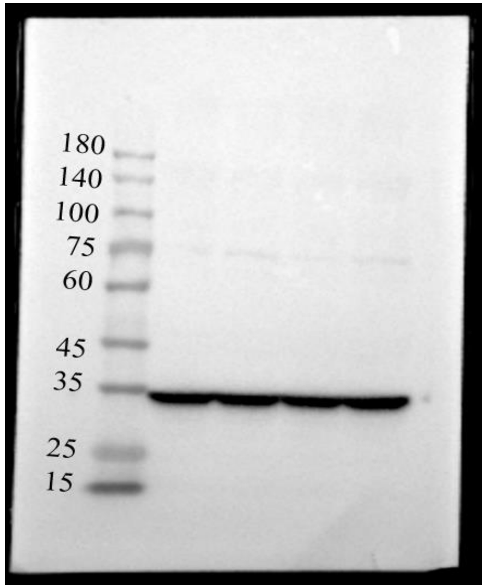

GAPDH (37KD) in Figure 5C

Figure S3: Flow cytometry diagram in Fig. 3A

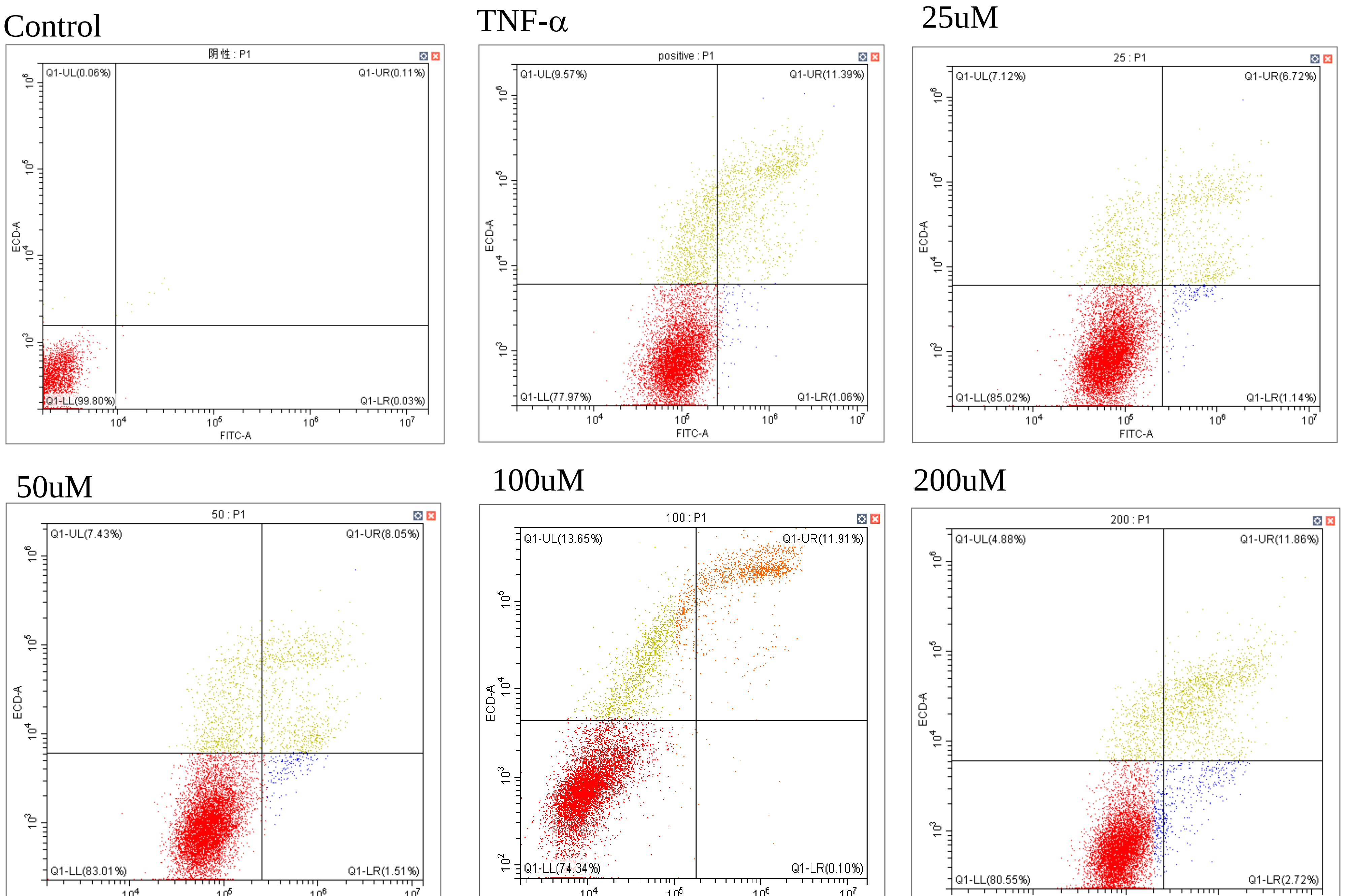

Figure S4: Flow cytometry diagram in Fig. 3B

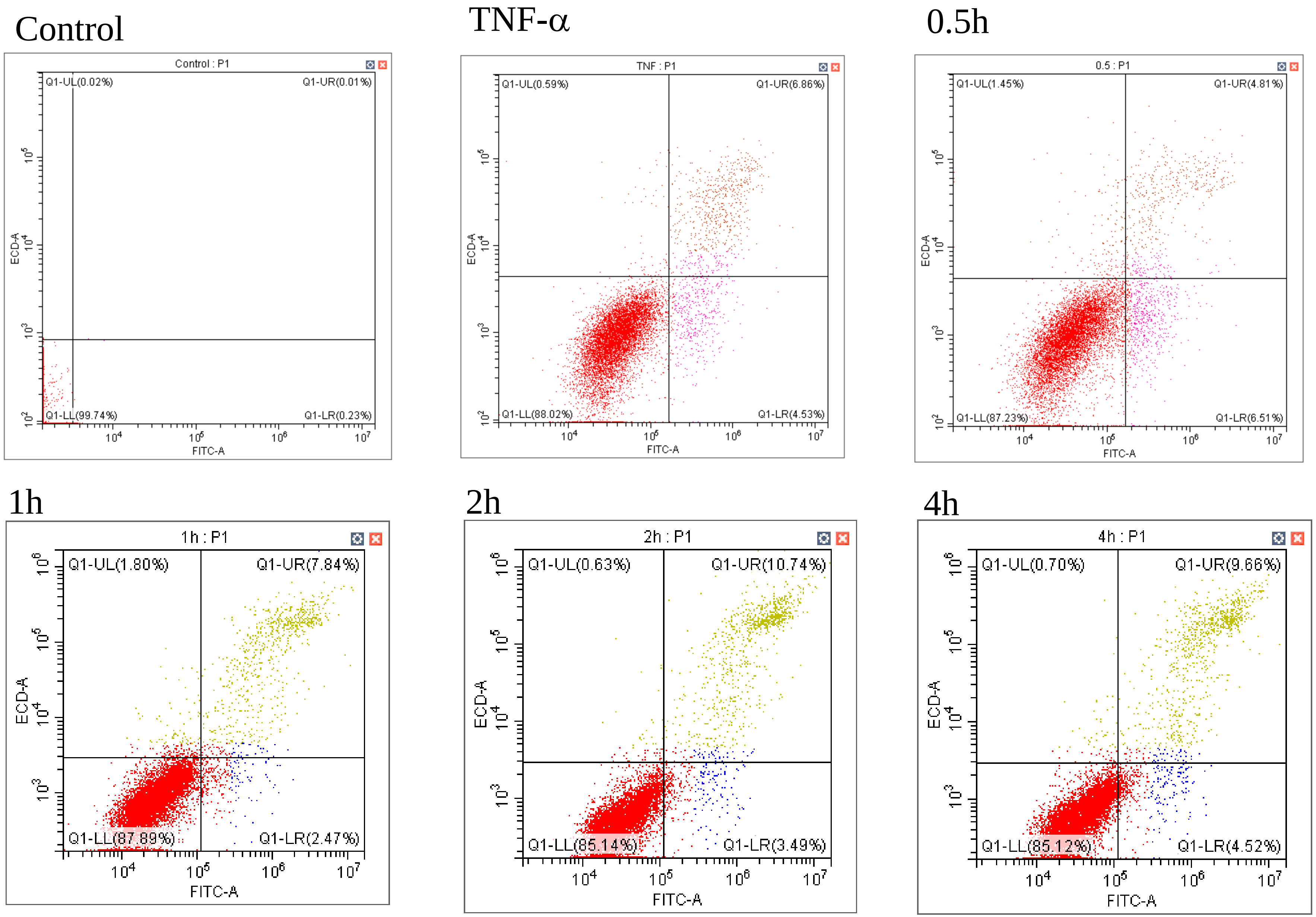

Figure S5: Flow cytometry diagram in Fig. 3C

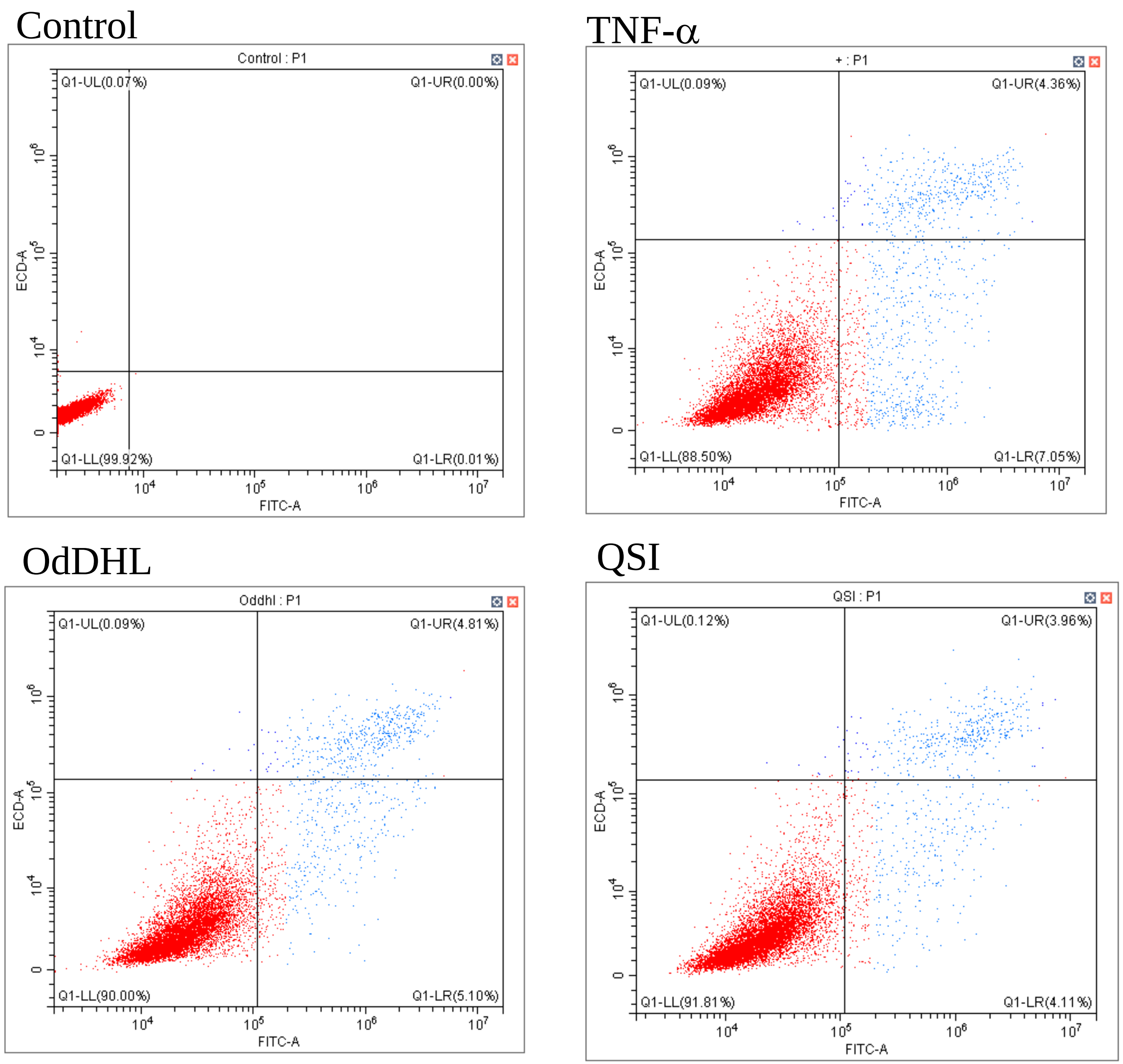

Figure S6: Flow cytometry diagram in Fig. 5F

Control

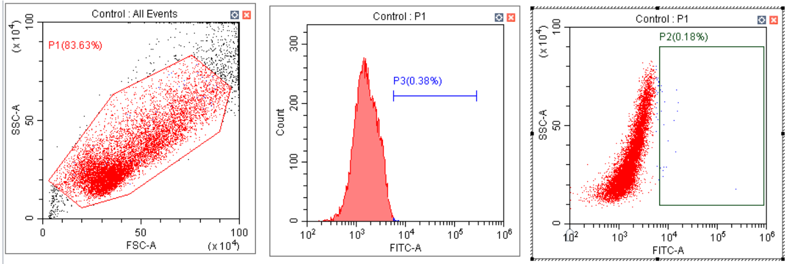

试管名称: Control
样本ID:

群体	颗粒数	%总数	%父群	Mean FITC-A
All Events	12000	100.00%	100.00%	2677.8
P1	10035	83.63%	83.63%	1833.9
P2	18	0.15%	0.18%	22032.2
P3	38	0.32%	0.38%	13570.4

OdDHL

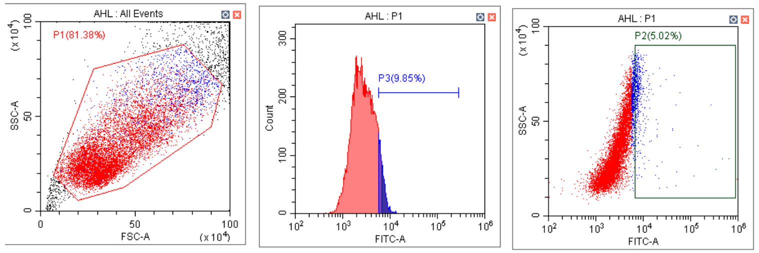

试管名称: AHL
样本ID:

群体	颗粒数	%总数	%父群	Mean FITC-A
All Events	12288	100.00%	100.00%	5290.3
P1	10000	81.38%	81.38%	3492.5
P2	502	4.09%	5.02%	12652.2
P3	985	8.02%	9.85%	8023.6

MβCD

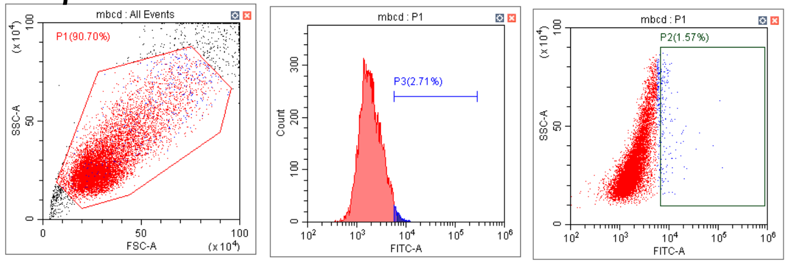

试管名称: mbcd
样本ID:

群体	颗粒数	%总数	%父群	Mean FITC-A
All Events	11025	100.00%	100.00%	2964.4
P1	10000	90.70%	90.70%	2276.9
P2	157	1.42%	1.57%	11210.4
P3	271	2.46%	2.71%	9027.9

Chol

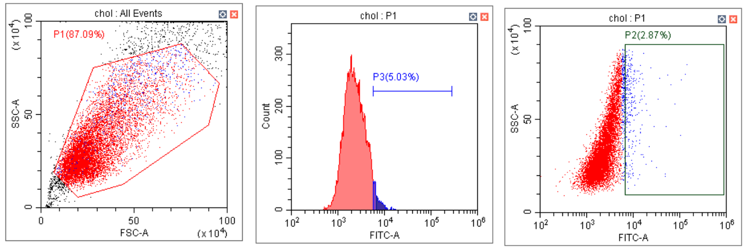

试管名称: chol
样本ID:

群体	颗粒数	%总数	%父群	Mean FITC-A
All Events	11483	100.00%	100.00%	3623.7
P1	10000	87.09%	87.09%	2763.7
P2	287	2.50%	2.87%	12036.5
P3	503	4.38%	5.03%	9470.8
